# Evaluation of Blood Pressure Control using a New Arterial Stiffness Parameter, Cardio-ankle Vascular Index (CAVI) 

**DOI:** 10.2174/1573402111309010010

**Published:** 2013-02

**Authors:** Kohji Shirai, Junji Utino, Atsuhito Saiki, Kei Endo, Masahiro Ohira, Daiji Nagayama, Ichiro Tatsuno, Kazuhiro Shimizu, Mao Takahashi, Akira Takahara

**Affiliations:** 1 Department of Vascular Function, Sakura Hospital, School of Medicine, Toho University, Chiba, Japan; 2 Diabetes Endocrine and Metabolism Center, Sakura Hospital, School of Medicine, Toho University, Chiba, Japan; 3 Cardiovascualr Center, Sakura Hospital, School of Medicine, Toho University, Chiba, Japan; 4 Mihama Hospital, Chiba, Japan, Faculty of Pharmaceutical Sciences, Toho University, Chiba, Japan; 5 Department Pharmacology and Therapeutics, Faculty of Pharmaceutical Sciences, Toho University, Chiba, Japan

**Keywords:** Cardio-ankle vascular index, arterial stiffness, angiotensin II receptor blockers, calcium channel blocker, hypertension.

## Abstract

Arterial stiffness has been known to be a surrogate marker of arteriosclerosis, and also of vascular function. Pulse wave velocity (PWV) had been the most popular index and was known to be a predictor of cardiovascular events. But, it depends on blood pressure at measuring time. To overcome this problem, cardio-ankle vascular index (CAVI) is developed. CAVI is derived from stiffness parameter *β* by Hayashi, and the equation of Bramwell-Hill, and is independent from blood pressure at a measuring time. Then, CAVI might reflect the proper change of arterial wall by antihypertensive agents.

CAVI shows high value with aging and in many arteriosclerotic diseases and is also high in persons with main coronary risk factors. Furthermore, CAVI is decreased by an administration of α_1_ blocker, doxazosin for 2-4 hours, Those results suggested that CAVI reflected the arterial stiffness composed of organic components and of smooth muscle cell contracture. Angiotensin II receptor blocker, olmesartan decreased CAVI much more than that of calcium channel antagonist, amlodipine, even though the rates of decreased blood pressure were almost same. CAVI might differentiate the blood pressure-lowering agents from the point of the effects on proper arterial stiffness.

This paper reviewed the principle and rationale of CAVI, and the possibilities of clinical applications, especially in the studies of hypertension.

## INTRODUCTION

The significance of arterial stiffness for the prognosis of cardiovascular diseases is nearly established [1-4]. Arterial stiffness is based on the structural changes occurring prior to plaque or thrombus formation in muscular and elastic vessels. Several methods have been designed to assess arterial stiffness including pulse wave velocity (PWV) [1-7] and augmentation index [8]. As for PWV, there were many methods such as carotid-femoral PWV (cfPWV) [9], heart to femoral PWV(hfPWV)[10] and brachial-ankle pulse wave velocity (baPWV) [11]. And, many data as a surrogate marker of arteriosclerosis had been reported [3-5, 12-15]. However, PWV is known to depend on blood pressure at the time of measurement [16, 17]. Then, the real effects of blood pressure control on the properties of arterial wall had not been accurately evaluated. 

In1980, Hayashi *et al*. [18] proposed the stiffness parameter β = 1n(Ps/Pd).D/∆D, where Ps is systolic, Pd is diastolic blood pressure, D is diameter of the artery, and ∆D is the change in arterial diameter according to blood pressure difference. This value does not depend on the blood pressure, theoretically. Kawasaki *et al*. [19] tried to measure stiffness parameter β in cervical artery using the echo-phase tracking system. A limitation of the stiffness parameter β is that it is applicable to a local segment of the artery. The cardio-ankle vascular index (CAVI) was developed to measure proper arterial stiffness with some length, according to the theory of stiffness parameter β. This time, CAVI was applied to the artery from the origin of the aorta to the ankle of tibial artery as shown in Fig. (**1**) [20]. The rationale for the expansion of stiffness β theory at one segment of the artery to some length of the artery composed of various types such as elastic artery and muscular artery, has to be confirmed from various aspects. Until now, many aspects of clinical studies on CAVI seemed to support the rightness of expanding β theory to some length of artery, as shown in Table **1** [21-26].

Especially, the studies using β_1_-aderenoceptor blocker and α_1_-aderenoceptor blocker indicated that CAVI was independent of blood pressure at measuring time, and reflected not only organic stiffness, but also the functional stiffness composed of smooth muscle contracture [24]. 

This review described the principle of CAVI and reviewed the recent reports about CAVI, focusing on the roles of CAVI in hypertension research. 

## 1. THE PROPERTIES OF CAVI 

### 1) The Principle of CAVI

**Fig. (1):**
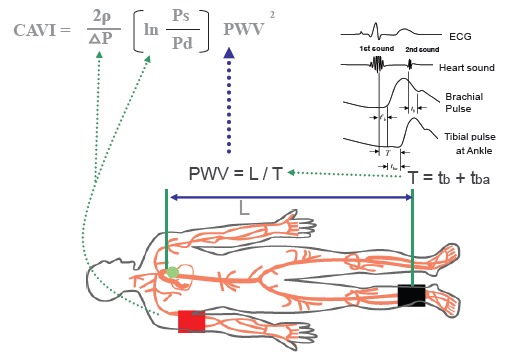
**CAVI and its measuring method (Ref. 20). **PWV from the heart to the ankle is obtained by measuring the length from the origin of the aorta to the ankle, and by calculating T = t_b_ + t_ba_. Blood pressure is measured at the brachial artery. Ps: systolic blood pressure, Pd: diastolic blood pressure, PWV: pulse wave velocity, ∆P: Ps - Pd, ρ: blood density, ∆P: pulse pressure, L: length from the origin of the aorta to the ankle, T: time taken for the pulse wave to propagate from the aortic valve to the ankle, t_ba_: time between the rise of brachial pulse wave and the rise of ankle pulse wave, t_b_: time between aortic valve closing sound and the notch of brachial pulse wave, t’_b_: time between aortic valve opening sound and the rise of brachial pulse wave.

The CAVI reflects the stiffness of the whole arterial segment comprising the aorta, femoral artery and tibial artery (Fig. **1**, from ref.^20^). This index was originally derived from the stiffness parameter β proposed by Hayashi [18] and Kawasaki *et al*. [19], and was expanded to some length of the artery with application of modified Bramwell-Hill’s equation [27].

CAVI = a{(2ρ/∆P) x ln(Ps/Pd) PWV^2^} + b ------ CAVI formula

where, Ps is systolic blood pressure, Pd is diastolic blood pressure, PWV is pulse wave velocity from the origin of the aorta to tibial artery at the ankle through the femoral artery, ∆P is Ps - Pd, ρ is blood density, and a and b are constants. 

Theoretically, blood pressure applied must be obtained at each every point from the origin of the aorta to tibial artery at the ankle. The problem is that the blood pressure is increasing from the origin of the aorta to the femoral artery, and is decreasing from the femoral artery to peripheral artery. Conveniently, mean blood pressure of the whole artery could be applied. In place of a mean blood pressure of the whole artery, blood pressure at the brachial artery was applied in CAVI. 

Thus, CAVI is originated from the stiffness parameter β = ln(Ps/Pd) . (D/∆D). D/∆D is calculated from PWV of some length of the artery and ∆P, in place of diameter change (D/∆D) [27]. 

### 2) The Rationales of CAVI to be Applied to some Length of Artery and of Independence from Blood Pressure at a Measuring Time

There still remain several questions for CAVI. One is whether it is valid to apply Bramwell–Hill’s equation to the equation of the stiffness parameter β, which is essentially applied to some segment of the artery. Takaki *et al*. [21] provided the evidence for the validity of CAVI by showing a positive correlation between the stiffness parameter β of the aorta and CAVI (r = 0.67, P <0.01). 

Next question is that, the most conspicuous feature of CAVI is theoretical independence of the blood pressure at the time of measurement. However, this has to be proven, experimentally. Several reports [21-24] showed that CAVI is less dependent on blood pressure than PWV. But, these results do not necessarily mean that CAVI is independent of blood pressure at the time of measurement.

We tried to solve this question using a selective β_1_ receptor blocker, metoprolol as shown in Fig. (**2A**) [24]. When metoprolol was administered to 12 men, systolic and diastolic blood pressure decreased for 6 hours. baPWV decreased accompanying with a decrease in blood pressure as expected, but CAVI did not change. This result indicated that CAVI was not influenced by blood pressure at the time of measurement. 

Then, CAVI could be used to evaluate the effect of blood pressure control on the proper stiffness of arterial wall. 

### 3) CAVI as a Surrogate Marker of Arteriosclerotic Diseases and Coronary Risk Factors

#### 1) Coronary Arterial Diseases

CAVI of healthy people in Japan without cardiovascular risk factors increases with aging from 20 to 70 years [25]. CAVI of men is higher than that of women in all ages by 0.2. 

As for coronary artery disease, CAVI increases as the number of coronary vessels with stenosis (>75%), increases [26]. The cutoff point of CAVI for the presence of coronary stenosis was 8.91 among the patients with a suspicion of ischemic coronary artery disease. Izuhara *et al*. [28] also reported the multiple logistic analysis revealing that CAVI, but not baPWV was associated with the presence of carotid and coronary arteriosclerosis. And several worker reported that CAVI was high in coronary artery disease [28-31]. 

Sairaku *et al*. [31] reported that CAVI was high in acute coronary syndrome and decreased after 6 months. Transient high CAVI might induce or trigger the acute coronary syndrome. 

#### 2) Carotid Arteriosclerosis

As for intima-media thickness (IMT) of carotid artery, several workers showed strong correlation between CAVI and IMT, but, plaque score showed much more stronger correlation with CAVI [27-30]. 

The combination of CAVI and IMT might be a much significant predictor of cerebral thrombosis in highly atherosclerotic patients.

#### 3) Chronic Kidney Diseases

As for chronic kidney disease, there were several reports that CAVI correlated with estimated glomerular filtration rate [32, 33]. CAVI is high in patients taking hemodialysis therapy [34, 35]. 

#### 4) Metabolic Syndrome

CAVI was high in metabolic syndrome. And, body weight reduction improved CAVI in obese patients on metabolic syndrome as well as reduction of risk factors [36].

#### 5) Cerebral Infarction

CAVI was related with cerebrovascular accidents [37].

#### 6) Coronary Risk Factors (Diabetes Mellitus, Dyslipidemia, Obesity)

CAVI is reported to be high in patients with diabetes mellitus [28, 29, 30, 38, 39]. Recent studies have shown that insulin therapy decreases CAVI, accompanying with lowered blood glucose level [36]. Glimepiride decreased CAVI accompanied with improved glucose level [40]. CAVI decreased by diphasic insulin aspart30/70[41]. 

CAVI is not so closely related with hypercholesterolemia as with diabetes mellitus [28, 39, 54]. However, some reports showed that CAVI is related to LDL-cholesterol level and also to cholesterol/HDL- cholesterol ratio [21]. Soska *et al*. [42] reported that CAVI was not necessarily high in heterozygous familiar hypercholesterolemic patients. Initial lipidosis induced by infiltration of LDL might soften the arterial wall, and when the complicated lesion occurred, CAVI might increase. Cholesterol lowering agents such as pitavastatin [43], ezetimibe [44] and triglyceride-lowering agent, eicosapentaenoic acid [45] were reported to decrease CAVI. 

#### 7) Weight Control and Others

Weight reduction through diet and exercise therapy over a 3-month period decreased CAVI values, significantly [36]. CAVI was improved by weight reduction using formula diet [46].

CAVI was high in smoking group, and decreased by stopping smoking [23, 47] CAVI was reported to be elevated in the patients with sleep apnea syndrome [48] and decreased by continuous positive airway pressure treatment [49]. 

Above results might suggest that the CAVI could be a good maker of arterial stiffness, and also is a marker of controlling the coronary risk factors (Refer to Tables **1**,**2**). 

Kubota [50] showed that high CAVI group (10>) showed high incidence of heart disease and cerebrovascular accidents in 3 years.

## 2. CAVI AS A MARKER OF SMOOTH MUSCLE CELL CONTRACTURE 

The factors regulating CAVI were composed not only of organic stiffness such as collagen or elastin, but also of smooth muscle cell contracture. Those were shown by the studies using α_1_-aderenoceptor blocker, doxazosin, which is a dilator of peripheral artery as shown in Fig. (**2**,**B**) [25]. CAVI was decreased as blood pressure decreased for about 4 hours, when doxazosin was administered to healthy men, as stated before. 

CAVI was reportedly decreased by anesthesia, although not significantly [51]. It is interesting that vasomoter nerve function influencing the stiffness of arterial wall could be monitored with CAVI. 

Beraprost sodium (beraprost) is active prostaglandin I_2_(PGI_2_) analogue with vasodilatory, antiplatelet and cytoprotective effects. Recently, Takahashi reported that beraprost sodium, PGI_2_ analogue, decreased CAVI without blood pressure lowering effect as shown in Fig. (**3**). [52].

CAVI was correlated with flow-mediated dilatation of the artery [53]. Those results might indicate that CAVI is reflecting endothelial dysfunction, in which NO synthesis is disturbed. 

Above these results support the idea that CAVI reflects the contracture of arterial smooth muscle cells controlled by cathecholamines, prostacyclin (PGI_2_), and NO synthesis in endothelial cells, in addition to organic stiffness. 

## 3. BLOOD PRESSURE AND CAVI

### 1) Hypertension and CAVI in Clinical Observation (Table 1)

CAVI is not affected by blood pressure at the time of measurement [20, 24, 57]. Therefore, the real effect of blood pressure on the proper arterial wall stiffness can be evaluated with CAVI. 

There were many papers reporting that CAVI showed high values in patients with hypertension [36, 38, 54-56] These reports were the first to demonstrate that the real effect of hypertension on the stiffness of arterial wall. But, there were also a few reports describing that there was no correlation between CAVI and blood pressure [30, 57]. And the contradictory results were obtained only by using CAVI, not by PWV. 

Interestingly, when sunitinib maleate, which is interferes with the growth of cancer cells and raises blood pressure as a side effect, was administered to a patient, the increase of CAVI was observed before blood pressure increased [58]. This finding suggests that CAVI may reflect the stress to the artery induced by sunitinib maleate before hypertension occurs. Recently, Yoshida reported that CAVI was high in pregnant women complicated with preeclampsia [59]. This report also suggested that CAVI monitoring in pregnant women may be helpful to detect the preeclampsia. 

### 2) Antihypertensive Agents and CAVI

Several papers reported the effect of antihypertensive agents on CAVI (Table **3**). The decreasing rates of blood pressure by those agents are not necessarily be correlated with improvement rate of CAVI.

#### (1) Calcium Channel Blockers

Calcium channel blocker (CCB) is one of the most useful antihypertensive agents. However, it is known that some of CCB have the activating action of the renin-angiotensin system (RAS) or catecholamine system as an unfavorable characteristic. And, there are several types such as L-channel blocker type. T-channel blocker type and N-channel blocker type. Amlodipine is known to be a L-channel blocker type. Kurata *et al*. reported that amlodipine decreased CAVI ( n= 10, 24 week) [60]. Miyashita *et al*. reported that the decrease by amlodipine was a little, and not significant [61]. 

**Fig. (4):**
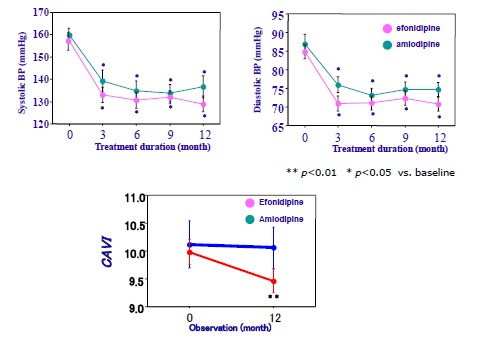
**The effects of efonidipine and amlodipine on Blood pressure and CAVI (Ref 62).** Efonidipine and amlodipine were CCBs. As shown in upper panel, blood pressures were decreased at almost same ratios. But, CAVI decreased much more by efonidipine than amlodipine.

Sasaki *et al*. [62] compared the effects of efonidipine, T-channel blocker, and of amlodipine (L-channel blocker). The blood pressures were reduced by almost same rates (Fig. **4**). But, CAVI was significantly reduced by efonidipine, but not by amlodipine. So, it is suggested that various types of Ca channel blockers had their own properties on arterial wall stiffness. 

It is very interesting point whether CAVI can differentiate the effects of different types of calcium channel blockers or not.

#### (2) Angiotensin II Receptor Antagonists and Angiotensin Converting Enzyme Inhibitors

Renin angiotensin aldosterone system is very important regulator of blood pressure. As for angiotensin II receptor antagonists (ARB), there were several reports. Telmisartan were reported to decrease CAVI [63]. Uehara [64] reported that candesartan reduced CAVI most among candesartan, telmisartan and losartan. Bukuda [65] studied the effect of candesartan comparing with calcium channel blockers (CCBs). They showed that blood pressure decreased significantly in both groups and the rates were not different in both group, but, candesartan significantly reduced CAVI, but not CCBs. These results were almost consistent with the studies on amlodipine and olmesartan by Miyashita *et al*. [61]. The blood pressures were decreased at same rate, but CAVI was decreased significantly only by olmesartan. 

As for prognosis of the treatments for hypertension, angiotensin 2 receptor antagonists or angiotensinogen converting enzyme inhibitor is reported to have better effect on the prognosis of cardiovascular diseases than calcium channel blockers [66]. The coincidence of the superiority of ARB to CCBs in the prospective mass study for long term, and in simple study using CAVI for short time, is interesting and important. Much attention should have to be paid to the effects of antihypertensive agents on CAVI in terms of the prognosis of cardiovascular events. 

#### (3) Thiazide

Diuretics are known to decrease blood pressure, but may exacerbate insulin resistance. Real effects of diuretics on arterial wall stiffness have not been done. There were no reports available to obtain the conclusion.

But, the combination of olmesartan and azelnidipine has advantages over the combination of olmesartan and thiazide with respect to CAVI in patients with moderate hypertension [67]. It might suggest that azelnidipine improved CAVI, but thiazide did not change CAVI. 

A tablet combining losartan and hydrochlorothiazide has been found to decrease CAVI [68]. Both agents might compensate each other to improve CAVI. 

#### (4) Other Antihypertensive Agents (α Blocker, β Blocker, Spironolactone)

Now, there were no reports available concerning the effects of α blocker, β blocker, and spironolactone on CAVI for long term. But, above data might suggest that CAVI might discriminate the effects of those antihypertensive agents on arterial stiffness in addition to blood pressure itself. 

## CONCLUSION

### Future of CAVI in the Hypertension Research - (Fig. 5)

It is almost established that CAVI is reflecting the arteriosclerotic change, and also smooth muscle cell contracture. Blood pressure control system were involving many factors such as cardiac output, arterial compliance and circulating blood volume. Among them, CAVI might be reflecting compliance. The analysis of the pathogenesis of hypertension and also the prognosis of the hypertension control might be more adequately evaluated with monitoring CAVI. 

CAVI may become useful index to evaluate the real effects of blood pressure control on the arteries. 

## Figures and Tables

**Fig. (2):**
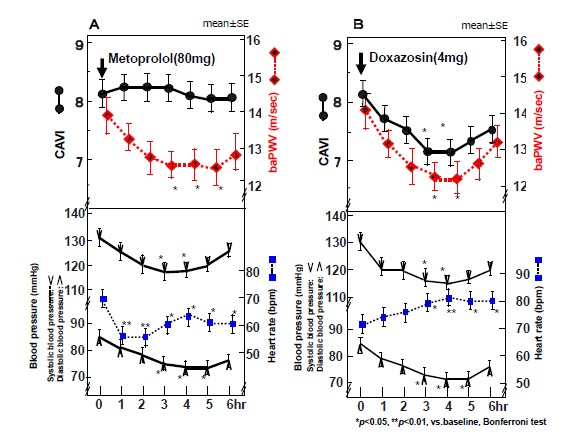
**Effects of the β_1_- blocker, metoprolol and α_1_- blocker, doxazosin on CAVI and baPWV (Ref. 24). **When selective β_1_ -adrenergic blocker metoprolol (80 mg) was administered, both systolic and diastolic blood pressures decrease and baPWV also decreases, but CAVI does not change (24) (**A**). This study indicates that CAVI is independent of blood pressure at the time of measurement. Furthermore, with the administration of selective α_1_-aderenergic receptor blocker, doxazosin, both systolic and diastolic blood pressures decreased and CAVI decreased as well as baPWV (**B**), indicating that CAVI decreased with a relaxation of smooth muscles induced by α_1_-aderenergic receptor blocker.

**Fig. (3):**
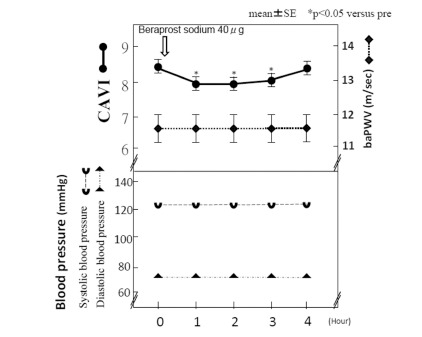
**Effects of beraprost administration on CAVI(Ref 52).** Vasodilator beraprost(40 *µ*g) was orally administered, and CAVI and blood pressure was measured every hour. Blood pressure was not changed, but CAVI decreased, but baPWV was not. These results suggested that CAVI reflected smooth muscle cell contracture, independently from blood pressure.

**Fig. (5):**
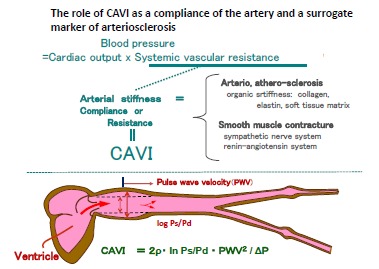
**The role of CAVI as a resistance or compliance of the artery and a surrogate marker of arteriosclerosis. ** CAVI might reflect the resistance or compliance of the artery, in addition to arteriosclerotic stiffness. Namely, CAVI reflects the vascular function, which compensates the heart function and promotes peripheral blood flow as a Windkessel. This might protect or improve left ventricular function (Ref^55^), and maintain the steady peripheral blood flow.

**Table 1.** CAVI in Arteriosclerotic Diseases and in Coronary Risks

Agents	CAVI Value	Reference

Aging, man > woman	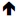	Namekata(38), Takaki(21)

Coronary artery diseases	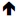	Nakamura(26), Izuhara(28), Miyoshi(29), Horinaka(30)
[Acute coronary disease]	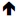	[Sairaku(31)]

Intima-media thickness of cervical artery	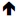	Nakamura(26), Izuhara(28), Miyoshi(29), Horinaka(30)

Chronic kidney disease	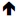	Kubozono(32), Nakamura (33), Satoh-Asahara(36)
Hemodialysis	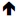	Ueyama(34), Ichihara(35)

Cerebral infarction	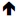	Suzuki(37)

Metabolic syndrome	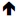	Satoh-Asahara(36)

Diabetes mellitus	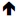	Namekata(38), Ibata (39), Izuhara(28)

Dyslipidemia	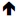	Takaki(21)

Smoking	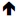	Kubozono(32), Noike(47)

Obstructive sleep apnea syndrome	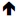	Kumagai(48), Kasai(49)

Hypertension	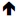	Kubozono(23), Satoh-Asahara(36), Namekata(38), Ibata(39), Okura(54), Sakane(55), Kadota(56), Takaki(57)
	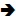	Horinaka(30)

**Table 2.** Improving Factor or Treatment for CAVI

Treatments	CAVI Value Change	Reference

Weight reduction	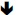	Satoh(36), Nagayama (46)

Blood glucose control	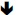	Nagayama(40), Ohira(41)

Lipid lowering agents		
Statin	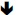	Miyashita(43)
Ezetimib	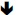	Miyashita(44)
Eichosapentanoic acid	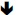	Satoh(45)

Stop smoking	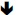	Noike(47)

Continuous pulmonary assisting	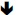	Kasai(49)

**Table 3.** Anti-hypertensive Agents and CAVI

Agents	CAVI value	Reference

Calcium channel blocker		
L type amlodipine	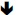 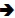	Kurata(60), Miyashita (61)
N type silnidipine	U.D	
T type efonidipine	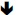	Sasaki(62)

Angiotensin 2 receptor blocker	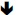	Kinouchi(63), Uehara(64), Miyashita(61), Bokuda(65)

Diuretics	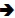	Ishimitsu (67), Kinouchi(68)

Spironolactone	U.D	

U.D: undetermined
